# Influence of High-Risk Pathological Factors and their Interaction on the Survival Benefit of Adjuvant Chemotherapy in Stage II Rectal Cancer: A Retrospective Study

**DOI:** 10.7150/jca.95769

**Published:** 2024-05-05

**Authors:** Kailong Zhao, Hongzhou Li, Wenwen Pang, Xuanzhu Zhao, Baofeng Zhang, Zhiqiang Fen, Leixin Jin, Jun Xue, Tianhao Chu, Suying Yan, Wanting Wang, Qiurong Han, Yao Yao, Xipeng Zhang, Xiaomin Su, Chunze Zhang

**Affiliations:** 1 School of Medicine, Nankai University, Tianjin, China.; 2 Department of Colorectal Surgery, Tianjin Union Medical Center, Tianjin, China.; 3 School of Integrative Medicine, Tianjin University of Traditional Chinese Medicine, Tianjin, China.; 4 Tianjin Medical University, Tianjin, China.; 5 Department of clinical laboratory, Tianjin Union Medical Center, Tianjin, China.; 6 The Institute of Translational Medicine, Tianjin Union Medical Center, Tianjin, China.; 7 Tianjin Institute of Coloproctology, Tianjin, China.; 8 Department of Gastroenterology, Tianjin Union Medical Center, Tianjin, China; 9 Department of Immunology, Nankai University School of Medicine, Nankai University, Tianjin, China.; 10 Department of General Surgery, The First Affiliated Hospital of Hebei North University, Hebei, China.; 11 Tianjin Academy of Traditional Chinese Medicine Affiliated Hospital, Tianjin, China.

**Keywords:** Rectal cancer, high-risk factors, interactions, adjuvant chemotherapy

## Abstract

**Objectives:** We investigated the impact of high-risk factors in stage II (TNM stage) rectal cancer patients to determine whether they benefit from adjuvant chemotherapy after surgery. Additionally, we explored the interaction between high-risk factors and adjuvant chemotherapy. Our study provides refined guidance for postoperative treatment in patients with stage II rectal cancer.

**Methods:** The retrospective study included 570 stage II rectal adenocarcinoma patients who underwent total mesorectal excision surgery at Tianjin Union Medical Center from August 2012 to July 2019. We employed Cox regression models to assess the collected pathological and clinical factors, identifying the risk factors for overall survival (OS) and disease-free survival (DFS). Additionally, we thoroughly examined the interaction between various high-risk pathological factors and postoperative chemotherapy (ACT), including multiplicative interaction (INTM) and additive interaction (RERI).

**Results:** Among the 570 stage II rectal cancer patients in this study, the average age was 62 years, with 58.9% (N=336) of the population being older than 60. Males accounted for the majority at 64.9% (N=370). Age was found to have an impact on whether patients received adjuvant chemotherapy after surgery (P<=0.001).Furthermore, age (HR: 1.916, 95% CI: 1.158-3.173, P=0.011; HR: 1.881, 95% CI: 1.111-3.186, P=0.019), TNM stage (HR: 2.216, 95% CI: 1.003-4.897, P=0.029; HR: 2.276, 95% CI: 1.026-5.048, P=0.043), the number of lymph nodes cleared during surgery (HR: 1.968, 95% CI: 1.112-3.483, P=0.017; HR: 1.864, 95% CI: 0.995-3.493, P=0.045), and lymphovascular invasion (HR: 2.864, 95% CI: 1.567-5.232, P=0.001; HR: 3.161, 95% CI: 1.723-5.799, P<0.001) were identified as independent risk factors for patients' overall survival (OS) and disease-free survival (DFS). Moreover, the interaction analysis, both multiplicative and additive, revealed significant interactions between the number of lymph nodes cleared during surgery and the administration of adjuvant chemotherapy. For OS (HR for multiplicative interaction: 0.477, p=0.045; RERI: -0.531, 95% CI: -1.061, -0.002) and for DFS (HR for multiplicative interaction: 0.338, p=0.039; RERI: -1.097, 95% CI: -2.190, -0.005).

**Conclusions:** This study provides insights into the complex relationship between adjuvant chemotherapy (ACT) and survival outcomes in stage II rectal cancer patients with high-risk pathological factors. The findings suggest that the number of cleared lymph nodes plays a significant role in the efficacy of ACT and underscores the need for individualized treatment decisions in this patient population.

## Introduction

Rectal cancer is a disease that poses a serious threat to human health and quality of life, with its incidence showing a continuous upward trend 1, 2. Among diagnosed rectal cancer patients, approximately 70% of them are at stage II and III 3. For resectable rectal cancer, radical resection surgery is the preferred treatment option, and surgery has a clear survival benefit for rectal cancer patients 4. However, existing research and literature on whether adjuvant chemotherapy and radiotherapy after surgery can benefit stage II and III rectal cancer patients have significant uncertainty 5, 6. Although there is clear evidence that postoperative chemotherapy benefits survival in stage II colon cancer patients 7, 8, rectal cancer and colon cancer differ clinically and in their biological behavior 9. In the NCCN guidelines, for stage II rectal cancer patients with high-risk factors, adjuvant chemotherapy is recommended after surgery 10, however, existing research lacks strong evidence to suggest a corresponding selection between high-risk pathological factors and postoperative chemotherapy in patients with stage II rectal cancer. Therefore, while guidelines recommend chemotherapy for patients with high-risk factors, observational therapy remains a viable option to consider.

Several studies on the benefits of adjuvant chemotherapy in rectal cancer patients have shown contradictory conclusions, and there is limited research on various subgroups based on high-risk factors in rectal cancer patients. Furthermore, there has been a lack of analysis regarding the interaction between various subgroups and adjuvant chemotherapy. Our study provides valuable insights in this direction and offers an explanation for these discrepancies in previous research.

## Materials and Methods

### Study design dan data collection methods

This retrospective single-center study focused on patients diagnosed with stage II (AJCC TNM stage) rectal adenocarcinoma (10 cm above the anal verge) between August 2012 and July 2019, who underwent total mesorectal excision surgery (TME) at Tianjin Union Medical Center. The inclusion criteria for patient data were as follows: (1) The inclusion criteria encompassed individuals with stage II rectal adenocarcinoma (age ≥ 18 years, ≤ 80 years); (2) Preoperative chest, abdominal, and pelvic CT or MRI scans; (3) Confirmation of no tumor residue by CT or MRI at the first follow-up visit; (4) Absence of abnormal bleeding tendencies; (5) Patients who did not receive preoperative neoadjuvant therapy or palliative surgical resection; (7) No severe acute or chronic illnesses in the three months preceding the study, including myocardial infarction, stroke, congestive heart failure, gastrointestinal bleeding within one year prior to the study, diabetes, and uncontrolled infections within the three months preceding the study. High-risk stage II rectal cancer patients were subjected to six months of adjuvant chemotherapy, involving regimens such as FOLFOX, FOLFIRI, and CAPEOX.

### Data information

The following variables were extracted from patients' medical records: gender, age, and the administration of adjuvant chemotherapy (ACT). This study incorporated various pathological characteristics, including TNM stage, lymphovascular invasion (LVI), perineural invasion (PNI), microsatellite instability (MMR) status, and the quantity of lymph nodes cleared during surgical procedures. Specifically, perineural invasion was defined as the encirclement of at least one-third of the nerve circumference by cancer cells, which could infiltrate any of the three nerve layers: the outer nerve layer, perineurium, and endoneurium. All pathological parameters were sourced from pathology reports stored in the hospital information system and meticulously reviewed by senior pathologists. The primary outcome measure focused on the overall survival (OS) of rectal cancer patients, with the secondary endpoint concentrating on disease-free survival (DFS). Both survival measures were calculated in months.

### Data analysis methods

For normally distributed data, continuous variables were expressed using the mean and subjected to analysis using a t-test. Categorical variables were presented as numerical values (in percentages) and analyzed through either the chi-squared test or Fisher's exact test.

In subgroup analyses, we employed a Cox proportional hazards model to estimate hazard ratios (HRs) along with their corresponding 95% confidence intervals (CIs). This allowed us to evaluate the association between the administration of adjuvant chemotherapy (ACT) and both disease-free survival (DFS) and overall survival (OS). To explore interactions between various variables and ACT, we employed the R programming language. Multiplicative interactions were quantified by examining the HR and p-value of the interaction term. Additionally, we assessed additive interactions using the relative excess risk due to interaction (RERI). When the HR for multiplicative interaction was less than 1.0 or the additive interaction parameter was less than 0, it indicated that individuals with specific pathological features derived greater benefits from ACT compared to those without these features.

## Results

### Baseline Characteristics

**Figure 1:**
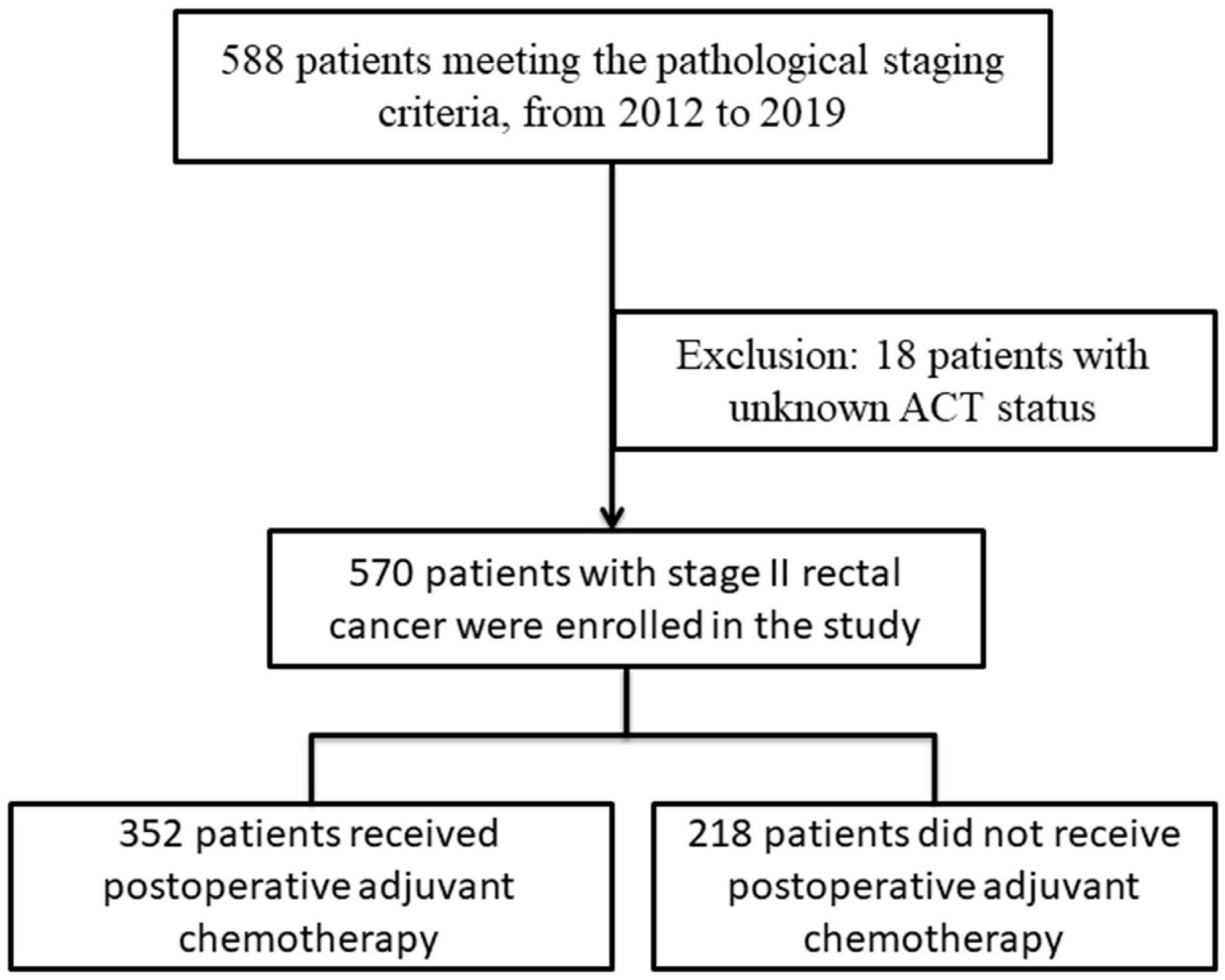
Patient Inclusion and Exclusion Flowchart.

In this study, 570 patients diagnosed with stage II rectal cancer were included. The process is shown in Figure 1. The average age of the participants was 62 years, with 370 (64.9%) being male and 200 (35.1%) female. The majority of patients were at IIA stage (526 individuals), while 53 patients had fewer than 12 lymph nodes removed during surgery. Among the participants, 352 received postoperative adjuvant chemotherapy, while 218 did not. Additionally, 47 patients exhibited vascular invasion, and 39 had neural infiltration. The results are presented in Table 1.

### Comparative Analysis of Clinical and Pathological Characteristics: ACT vs No ACT

In patients receiving ACT, there is a statistically significant difference in age, with a higher proportion of individuals under the age of 60 (31.7% vs. 46.9%, P < 0.001). However, there were no statistically significant differences between patients receiving ACT and those not receiving ACT in terms of gender, tumor TNM stage, number of lymph nodes cleared during surgery, neural infiltration, vascular invasion, and microsatellite status. The results are presented in Table 2.

### Univariate and Multivariate Cox Regression for Survival

The median follow-up time for all patients was 63 months. In the univariate Cox regression analysis, patients over the age of 60, those with IIB stage tumors, those with less than 12 lymph nodes cleared, and those with vascular invasion had worse overall survival (OS) with hazard ratios (HR) and 95% confidence intervals (CI) of 2.048 (1.241-3.370, P = 0.005), 2.431 (1.093-5.406, P = 0.049), 1.968 (1.112-3.483, P = 0.019), and 2.864 (1.567-5.232, P = 0.001), respectively. Similar results were observed in the univariate regression for disease-free survival (DFS), where these factors were associated with poorer DFS: HR95%CI: 2.051 (1.214-3.465, P = 0.007), HR95%CI: 2.171 (1.098-4.804, P = 0.054), HR95%CI: 1.873 (1.006-3.487, P = 0.048), and HR95%CI: 3.114 (1.704-5.690, P < 0.001). Additionally, in the univariate analysis, patients who received adjuvant chemotherapy had better OS and DFS, with p-values reaching statistical significance. The results are presented in Table 3.

To further analyze the data, factors with p-values less than or equal to 0.2 from the univariate analysis were included in the multivariate regression analysis. Age, tumor T stage, the number of lymph nodes cleared, and the presence of vascular invasion were found to be independent risk factors for overall survival (OS) with hazard ratios (HR) and 95% confidence intervals (CI) of 1.916 (1.158-3.173, P = 0.011), 2.431 (1.093-5.406, P = 0.029), 1.968 (1.112-3.483, P = 0.017), and 2.864 (1.567-5.232, P = 0.001). Similarly, in the multivariate analysis for disease-free survival (DFS), these factors were identified as independent risk factors with the following hazard ratios (HR) and 95% confidence intervals (CI): 1.881 (1.111-3.186, P = 0.019), 2.276 (1.026-5.048, P = 0.043), 1.864 (0.995-3.493, P = 0.045), and 3.161 (1.723-5.799, P < 0.001). The results are presented in Table 3.

### Survival Analysis and Interaction in Adjuvant Chemotherapy Subgroups by Clinical and Pathological Factors

**Figure 2:**
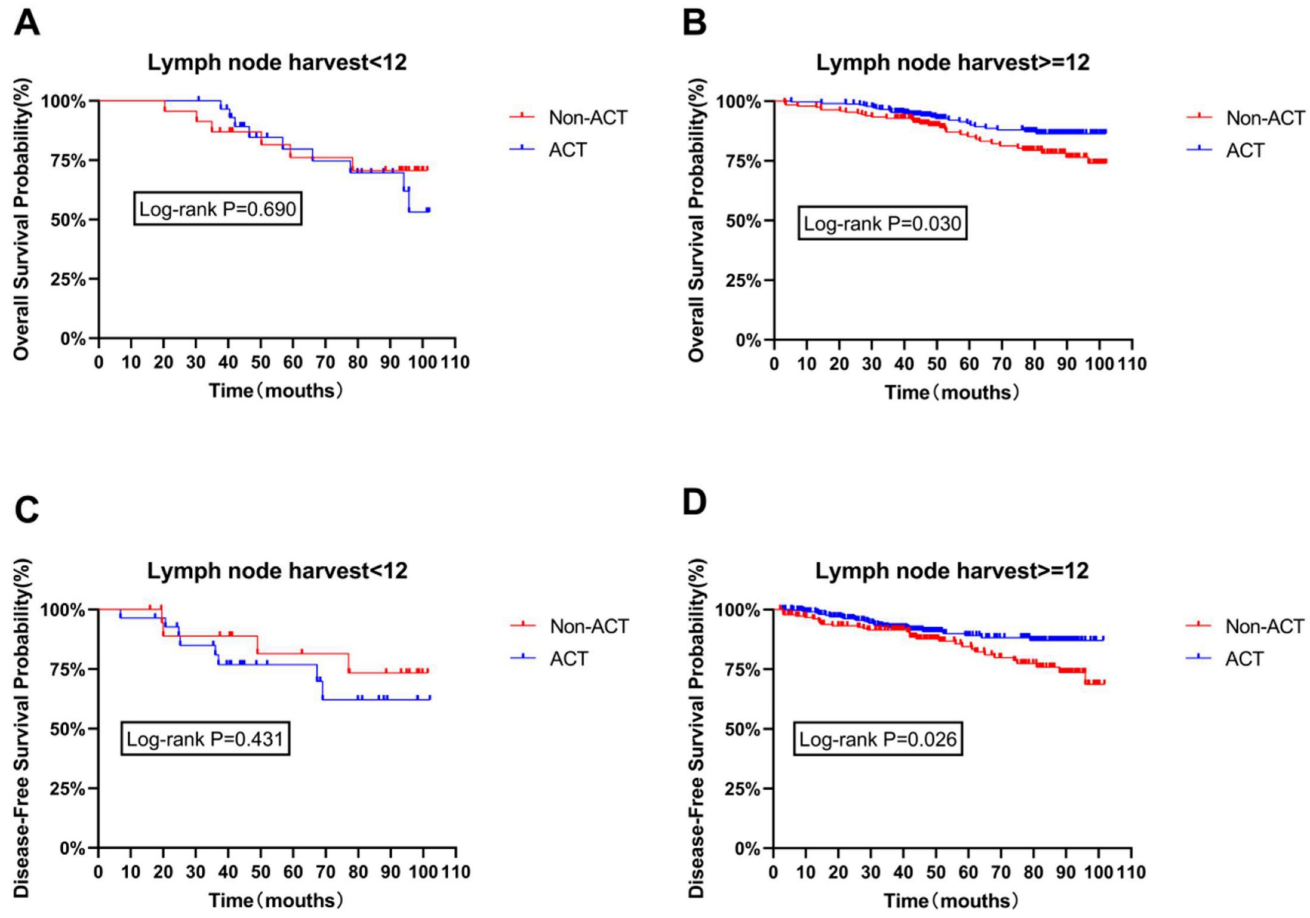
Disease-free survival curves of the subpopulation were calculated according to Kaplan-Meier method, and the use of ACT in patients with different number of lymph nodes.

The age difference between the ACT and non-ACT groups is significant, and age is recognized as an independent risk factor for both OS and DFS. While the other three survival risk factors did not exhibit significant statistical differences between the presence or absence of chemotherapy, they are still considered significant determinants of survival. Therefore, age, tumor staging (TNM stage), the number of lymph nodes cleared, and vascular invasion are considered potential confounding factors that could influence the impact of chemotherapy on OS and DFS. Subgroup survival analysis was conducted after adjusting for these factors. The results indicate that the number of lymph nodes cleared is a critical pathological factor affecting patient survival. Notably, in patients with 12 or more lymph nodes cleared, ACT significantly improved both OS and DFS (P=0.030, P=0.026), as shown in Figure 2B and Figure 2D. However, no significant statistical differences were found between ACT and non-ACT patients for the other included high-risk factors, as shown in Figure S1 and Figure S2 (Supplementary materials).

Furthermore, after adjusting the Cox risk model, it was observed that there was a statistically significant multiplicative interaction between lymph node clearance of 12 or more and ACT for OS (INTM: OR=0.477, P=0.045). This trend was confirmed in the additive interaction as well (RERI: -0.531, 95% CI: -1.061, -0.002). Please refer to Table 4 for details. Similarly, in terms of DFS, the same multiplicative and additive interactions were observed (INTM: OR=0.338, P=0.039; RERI: -1.097, 95% CI: -2.190, -0.005).

## Discussion

In this study, we conducted subgroup analysis based on the clinical high-risk pathological factors of stage II rectal cancer as outlined in the guidelines. After performing these subgroup analyses, we found that the number of lymph nodes cleared had a significant impact on the overall survival and disease-free survival with adjuvant chemotherapy (ACT). However, the other included pathological factors in the study did not exhibit an interaction with ACT, indicating that adjuvant chemotherapy did not significantly affect the survival of rectal cancer patients with or without these clinical high-risk pathological factors.

In the current NCCN guidelines, it is recommended that patients with stage II rectal cancer, particularly those with high-risk pathological factors, receive adjuvant chemotherapy (ACT) after curative surgery. However, there has been an ongoing debate regarding whether postoperative chemotherapy is necessary for stage II rectal cancer patients. While there has been relatively limited research on postoperative adjuvant chemotherapy for stage II rectal cancer in the past, some studies have indicated the presence of different clinical and pathological risk stratifications in stage II rectal cancer, which could impact patient prognosis 11. Furthermore, some studies have suggested that postoperative chemotherapy can significantly improve the survival of patients with stage II rectal cancer^12^. However, there are also studies indicating that postoperative chemotherapy does not have a significant impact on patient survival 13. A long-term survival analysis based on SEER data showed that adjuvant chemotherapy can improve the 5-year overall survival for stage II/III rectal cancer but does not enhance cancer-specific survival (CSS) 14. The inconsistency in research findings suggests that the choice of adjuvant chemotherapy for stage II rectal cancer may require more refined investigations into specific patient characteristics. In our study, the number of cleared lymph nodes demonstrated a significant influence on patient survival with ACT. Additionally, the effect of ACT on patient survival varied among different lymph node clearance levels.

Lymph node involvement is a crucial factor affecting the postoperative prognosis of rectal cancer 15, 16. In clinical practice, the number of lymph nodes examined and the extent of lymph node clearance in surgical specimens are used as indicators of lymph node involvement. Past research has consistently shown that the postoperative clearance of lymph nodes is significantly associated with the prognosis of stage II rectal cancer patients 17, 18. However, it's important to note that there's ongoing debate about the minimum number of lymph nodes that should be cleared. Some studies, such as Scott et al., recommend a minimum of 13 lymph nodes 19, while Hernanz et al. suggest at least 10 20. Tepper et al.'s research, on the other hand, suggests that a minimum of 14 lymph nodes is necessary to determine lymph node status in stage II rectal cancer patients 21. While this specific topic isn't the main focus of our study, we conducted a categorical statistical analysis based on the NCCN guidelines, which recommend a threshold of 12 lymph nodes.

Within our results in Figure 2, an intriguing observation emerges. Among patients with fewer than 12 cleared lymph nodes, those who received adjuvant chemotherapy (ACT) exhibit a tendency towards poorer overall survival (OS) and disease-free survival (DFS) compared to their non-ACT counterparts. However, this trend lacks statistical significance. In contrast, among patients with more than 12 cleared lymph nodes, a significant statistical difference favors ACT, indicating a better prognosis for these ACT patients.

Several factors can impact the number of cleared lymph nodes 22, including surgical technique 23, tumor location, extent of mesorectal excision, tumor size, cancer staging, patient-related factors (such as age and gender) 24, and the accuracy and experience of the pathologist 25. Notably, the immune status of the patient, particularly the microsatellite status, significantly affects the response to chemotherapy in stage II rectal cancer patients 26. Previous research has demonstrated that in cases of dMMR (microsatellite instability), chemotherapy based on 5-FU not only fails to provide benefits but can also lead to adverse reactions 27-29. This dMMR condition is most prevalent in stage II rectal cancer 30. Moreover, among the patients in our study who received ACT, the FOLFOX regimen was the most common, followed by the CapeOx regimen. Hence, the prevalence of dMMR and the choice of chemotherapy regimen could be contributing factors to the observed outcomes 31, 32.

Additionally, when patients have fewer than 12 cleared lymph nodes, it often indicates that the surgery may not have been well-tolerated. In such cases, surgeons tend to minimize the extent and duration of the procedure, suggesting that these patients may not tolerate postoperative chemotherapy, potentially leading to a worse prognosis for ACT patients compared to non-ACT patients.

To further substantiate our findings, we employed interaction analysis in medical statistics, which serves to validate our conclusions. Additionally, we conducted subgroup analyses for each individual factor within the high-risk features based on conventional statistical methods and interactions. We observed that previous studies predominantly focused on the concept of high-risk factors without further analysis. A large-scale study on high-risk factors and adjuvant chemotherapy in colon cancer found that adjuvant chemotherapy had an adverse effect on patient survival, regardless of whether it was a single high-risk pathological factor or multiple factors 33. However, a large retrospective analysis in Japan yielded contradictory results 34, 35. Most of these studies were based on extensive data containing numerous confounding factors. Therefore, our study endeavored to mitigate this limitation as much as possible and utilized interaction analysis, thereby providing stronger evidence for our results. In addition, the related contents of rectal cancer were also studied and explained.

Age is another potential contributor to this phenomenon, particularly in older patients (aged 70 or older). In this demographic, difficulties in tolerating surgery and a compromised immune status may lead to a lack of benefit from adjuvant chemotherapy.

However, for patients with more than 12 cleared lymph nodes, the benefits of ACT become evident. Therefore, for patients with stage II rectal cancer who have had more than 12 lymph nodes cleared during surgery, adjuvant chemotherapy is recommended.

Most prior research is based on large databases and the influence of confounding factors can significantly impact the results. Moreover, there has been limited research on the relationship between lymph nodes and stage II rectal cancer. In our study, to minimize the impact of other confounding factors, we explored the multiplicative and additive interactions of each included factor with OS and DFS. This approach further strengthens the validity of our conclusions and represents a notable advantage of our study.

As for other included clinical-pathological factors, such as tumor T stage, there is limited research on rectal cancer, but studies in colon cancer have been reported. For instance, Kumar et al.'s research suggested that in colon cancer, only patients with IIB tumors benefit from ACT in terms of OS and DFS 36. However, in our study, both IIA and IIB rectal cancer patients demonstrated better OS and DFS with ACT compared to non-ACT patients. While these differences were not statistically significant, they provide valuable directions for further research. Similar results were observed in the case of vascular invasion.

This study has several limitations. Firstly, it is a retrospective analysis, and the collection of patient information may be subject to recall bias. Secondly, there were significant differences in the proportions of certain factors within the study population, which could potentially impact the accuracy of the conclusions. Furthermore, the factors influencing chemotherapy regimens and survival benefits in stage II rectal cancer patients are not limited to those included in this study and require further exploration. However, the study also has several strengths. Firstly, there is limited research on rectal cancer, and our conclusions can provide valuable directions and references for further research in this area. Secondly, we used interaction analyses to quantitatively assess the relationship between factors and outcomes, which is a less common approach in previous studies. Thirdly, there is a scarcity of research specifically focused on rectal cancer patients, with most studies being based on colon cancer. However, there are biological differences between the colon and rectum. Therefore, our study contributes valuable findings in this area, offering clearer evaluation criteria for postoperative adjuvant therapy in rectal cancer patients.

## Conclusions

This study indicates that age, T stage, the number of lymph nodes cleared during surgery, and lymphovascular invasion are independent risk factors influencing overall survival and disease-free survival in patients with stage II rectal cancer. Furthermore, we observed an interaction between postoperative adjuvant chemotherapy and specific pathological factors, particularly in patients with a higher number of cleared lymph nodes, where adjuvant chemotherapy may confer greater survival benefits. These findings provide important clinical insights for guiding treatment decisions in stage II rectal cancer patients, emphasizing the significance of personalized therapy.

## Supplementary Material

Supplementary figures.

## Figures and Tables

**Table 1:** Baseline Characteristics

Clinical pathological features	Overall (N=570)	Percentage (%)
**Age**	62.0 [24.0, 87.0]	
<=60	234	41.1%
>60	336	58.9%
**Sex**		
Female	200	35.1%
Male	370	64.9%
**TNM stage**		
IIA	526	92.3%
IIB	44	7.7%
**Lymph node harvest**		
<12	53	9.3%
>=12	517	90.7%
**ACT**		
No	218	38.2%
Yes	352	61.8%
**VNI**		
No	523	91.8%
Yes	47	8.2%
**PNI**		
No	531	93.2%
Yes	39	6.8%
**MMR**		
dMMR	157	27.5%
pMMR	413	72.5%

ACT: Adjuvant Chemotherapy; LVI: Lymphovascular Invasion; PNI: Perineural Invasion; MMR: Microsatellite Instability; dMMR: Mismatch Repair Deficiency; pMMR: Protein Mismatch Repair Deficiency

**Table 2:** Stratification of Clinical and Pathological Factors for Adjuvant Chemotherapy

Name	Levels	Non-ACT(N=218)	ACT (N=352)	p
**Age**				
	<=60	69 (31.7%)	165 (46.9%)	<.001
	>60	149 (68.3%)	187 (53.1%)	
**Sex**				
	Female	83 (38.1%)	117 (33.2%)	0.278
	Male	135 (61.9%)	235 (66.8%)	
**TNM stage**				
	IIA	205 (94%)	321 (91.2%)	0.283
	IIB	13 (6%)	31 (8.8%)	
**Lymph node harvest**				
	<12	23 (10.6%)	30 (8.5%)	0.508
	>=12	195 (89.4%)	322 (91.5%)	
**VNI**				
	No	197 (90.4%)	326 (92.6%)	0.429
	Yes	21 (9.6%)	26 (7.4%)	
**PNI**				
	No	206 (94.5%)	325 (92.3%)	0.410
	Yes	12 (5.5%)	27 (7.7%)	
**MMR**				
	dmmr	58 (26.6%)	99 (28.1%)	0.766
	pmmr	160 (73.4%)	253 (71.9%)	

ACT: Adjuvant Chemotherapy; LVI: Lymphovascular Invasion; PNI: Perineural Invasion; MMR: Microsatellite Instability; dMMR: Mismatch Repair Deficiency; pMMR: Protein Mismatch Repair Deficiency

**Table 3:** Impact of Clinical and Pathological Factors on Survival: COX Univariate and Multivariate Regression Analysis

	OS				DFS			
	Univariate analysis HR (95% CI)	P	Multivariate analysis HR (95% CI)	P	Univariate analysis HR (95% CI)	P	Multivariate analysis HR (95% CI)	P
Age (>60)	2.048(1.241-3.370)	0.005	1.916(1.158-3.173)	0.011	2.051(1.214-3.465)	0.007	1.881(1.111-3.186)	0.019
Sex (Male)	0.990(0.624-1.570)	0.967			0.855(0.532-1.373)	0.517		
TNM stage (IIB)	2.216(1.003-4.897)	0.049	2.431(1.093-5.406)	0.029	2.171(0.981-4.804)	0.054	2.276(1.026-5.048)	0.043
LNH (<12)	1.967(1.117-3.464)	0.019	1.968(1.112-3.483)	0.017	1.873(1.006-3.487)	0.048	1.864(0.995-3.493)	0.045
VNI (yes)	2.692(1.482-4.890)	0.001	2.864(1.567-5.232)	0.001	3.114(1.704-5.690)	<0.001	3.161(1.723-5.799)	<0.001
PNI (yes)	1.590(0.765-3.300)	0.214			1.618(0.776-3.376)	0.200	1.251(0.572-2.732)	0.575
ACT (yes)	0.667(0.427-1.040)	0.074	0.703(0.449-1.100)	0.123	0.661(0.416-1.050)	0.080	0.723(0.454-1.150)	0.171
Mmr (pmmr)	0.962(0.574-1.620)	0.893			0.939(0.512-1.721)	0.838		

LNH: Lymph node harvest; LVI: LymphovascularInvasion; PNI: PerineuralInvasion; MMR: MicrosatelliteInstability; pMMR: Protein Mismatch Repair Deficiency; OS: OverallSurvival; DFS: Disease-FreeSurvival; HR: Hazard Ratio

**Table 4:** Subgroup Analysis of Pathological Factors for OS and DFS and Interaction with Adjuvant Chemotherapy

	ACT VS Non-ACT					
	OS			DFS		
	Multiplicative interaction	P for INTM	Additive interaction RERI (95% CI)	Multiplicative interaction	P for INTM	Additive interaction RERI (95% CI)
Age	0.4909	0.198	-1.430(-3.837, 0.975)	0.441	0.166	-1.679(-4.503, 1.146)
Sex	1.660	0.292	0.355(-0.233, 0.944)	1.859	0.208	0.446(-0.074, 0.965)
TNM	0.810	0.783	-0.876(-4.226, 2.474)	0.973	0.973	-0.478(-3.457, 2.500)
LNH	0.477	0.045	-0.531(-1.061, -0.002)	0.338	0.039	-1.097(-2.190, -0.005)
VNI	0.814	0.735	-0.164(-2.913, 2.586)	1.226	0.740	-0.163(-2.912, 2.585)
PNI	0.612	0.511	-0.952(-3.378, 1.473)	0.554	0.431	-1.169(-3.800, 1.463)
MMR	0.419	0.120	-1.014(-2.886, 0.856)	0.576	0.101	-1.619(-4.867, 1.629)

LNH: Lymph node harvest; LVI: Lymphovascular Invasion; PNI: Perineural Invasion; MMR: Microsatellite Instability; OS: Overall Survival; DFS: Disease-Free Survival; HR: Hazard Ratio; RERI: Relative Excess Risk due to Interaction; INTM: Multiplicative interaction

## Abbreviations

ACT: AdjuvantChemotherapy
OS: OverallSurvival
DFS: Disease-FreeSurvival
LVI: LymphovascularInvasion
PNI: PerineuralInvasion
MMR: MicrosatelliteInstability
DMMR: Mismatch Repair Deficiency
PMMR: Protein Mismatch Repair Deficiency
HR: Hazard Ratio
INTM: Rultiplicative Interaction
RERI: Relative Excess Risk due to Interaction
CSS: Cancer-specific survival

## References

[1] Benson AB 3rd, Bekaii-Saab T, Chan E, Chen YJ, Choti MA, Cooper HS (2012). Rectal cancer. J Natl Compr Canc Netw.

[2] Birkenkamp-Demtroder K, Olesen SH, Sørensen FB, Laurberg S, Laiho P, Aaltonen LA (2005). Differential gene expression in colon cancer of the caecum versus the sigmoid and rectosigmoid. Gut.

[3] Gray R, Barnwell J, McConkey C, Hills RK, Williams NS, Kerr DJ (2007). Adjuvant chemotherapy versus observation in patients with colorectal cancer: a randomised study. Lancet.

[4] Prolongation of the disease-free interval in surgically treated rectal carcinoma N Engl J Med. 1985; 312: 1465-72.

[5] André T, Boni C, Navarro M, Tabernero J, Hickish T, Topham C (2009). Improved overall survival with oxaliplatin, fluorouracil, and leucovorin as adjuvant treatment in stage II or III colon cancer in the MOSAIC trial. J Clin Oncol.

[6] Chen WW, Wang WL, Dong HM, Wang G, Li XK, Li GD (2022). The number of cycles of adjuvant chemotherapy in stage III and high-risk stage II rectal cancer: a nomogram and recursive partitioning analysis. World J Surg Oncol.

[7] André T, de Gramont A, Vernerey D, Chibaudel B, Bonnetain F, Tijeras-Raballand A (2015). Adjuvant Fluorouracil, Leucovorin, and Oxaliplatin in Stage II to III Colon Cancer: Updated 10-Year Survival and Outcomes According to BRAF Mutation and Mismatch Repair Status of the MOSAIC Study. J Clin Oncol.

[8] Booth CM, Nanji S, Wei X, Peng Y, Biagi JJ, Hanna TP (2016). Use and Effectiveness of Adjuvant Chemotherapy for Stage III Colon Cancer: A Population-Based Study. J Natl Compr Canc Netw.

[9] Lee YC, Lee YL, Chuang JP, Lee JC (2013). Differences in survival between colon and rectal cancer from SEER data. PLoS One.

[10] Benson AB, Venook AP, Al-Hawary MM, Azad N, Chen YJ, Ciombor KK (2022). Rectal Cancer, Version 2.2022, NCCN Clinical Practice Guidelines in Oncology. J Natl Compr Canc Netw.

[11] Chand M, Bhangu A, Wotherspoon A, Stamp GWH, Swift RI, Chau I (2014). EMVI-positive stage II rectal cancer has similar clinical outcomes as stage III disease following pre-operative chemoradiotherapy. Ann Oncol.

[12] Simillis C, Singh H, Afxentiou T, Mills S, Warren OJ, Smith JJ (2020). Postoperative chemotherapy improves survival in patients with resected high-risk Stage II colorectal cancer: results of a systematic review and meta-analysis. Colorectal Dis.

[13] Loree JM, Kennecke HF, Lee-Ying RM, Goodwin RA, Powell ED, Tang PA (2018). Impact of Postoperative Adjuvant Chemotherapy Following Long-course Chemoradiotherapy in Stage II Rectal Cancer. Am J Clin Oncol.

[14] Liao H, Zeng T, Xie X, Li J, Li D (2023). Adjuvant chemotherapy does not improve cancer-specific survival for pathologic stage II/III rectal adenocarcinoma after neoadjuvant chemoradiotherapy and surgery: evidence based on long-term survival analysis from SEER data. Int J Colorectal Dis.

[15] Newland RC, Chapuis PH, Smyth EJ (1987). The prognostic value of substaging colorectal carcinoma. A prospective study of 1117 cases with standardized pathology. Cancer.

[16] Wolmark N, Fisher B, Wieand HS (1986). The prognostic value of the modifications of the Dukes' C class of colorectal cancer. An analysis of the NSABP clinical trials. Ann Surg.

[17] Swanson RS, Compton CC, Stewart AK, Bland KI (2003). The prognosis of T3N0 colon cancer is dependent on the number of lymph nodes examined. Ann Surg Oncol.

[18] Tsai HL, Lu CY, Hsieh JS, Wu DC, Jan CM, Chai CY (2007). The prognostic significance of total lymph node harvest in patients with T2-4N0M0 colorectal cancer. J Gastrointest Surg.

[19] Scott KW, Grace RH (1989). Detection of lymph node metastases in colorectal carcinoma before and after fat clearance. Br J Surg.

[20] Hernanz F, Revuelta S, Redondo C, Madrazo C, Castillo J, Gómez-Fleitas M (1994). Colorectal adenocarcinoma: quality of the assessment of lymph node metastases. Dis Colon Rectum.

[21] Tepper JE, O'Connell MJ, Niedzwiecki D, Hollis D, Compton C, Benson AB 3rd (2001). Impact of number of nodes retrieved on outcome in patients with rectal cancer. J Clin Oncol.

[22] Scabini S, Montecucco F, Nencioni A, Zoppoli G, Sartini M, Rimini E (2013). The effect of preoperative chemoradiotherapy on lymph nodes harvested in TME for rectal cancer. World J Surg Oncol.

[23] El-Gazzaz G, Hull T, Hammel J, Geisler D (2010). Does a laparoscopic approach affect the number of lymph nodes harvested during curative surgery for colorectal cancer?. Surg Endosc.

[24] Altintas S, Bayrak M (2019). Assessment of Factors Influencing Lymph Node Count in Colorectal Cancer. J Coll Physicians Surg Pak.

[25] Tonini V, Birindelli A, Bianchini S, Cervellera M, Bacchi Reggiani ML, Wheeler J (2020). Factors affecting the number of lymph nodes retrieved after colo-rectal cancer surgery: A prospective single-centre study. Surgeon.

[26] Taieb J, Svrcek M, Cohen R, Basile D, Tougeron D, Phelip JM (2022). Deficient mismatch repair/microsatellite unstable colorectal cancer: Diagnosis, prognosis and treatment. Eur J Cancer.

[27] Zhao P, Ma YG, Zhao Y, Liu D, Dai ZJ, Yan CY (2019). MicroRNA-552 deficiency mediates 5-fluorouracil resistance by targeting SMAD2 signaling in DNA-mismatch-repair-deficient colorectal cancer. Cancer Chemother Pharmacol.

[28] Chan GHJ, Chee CE (2019). Making sense of adjuvant chemotherapy in colorectal cancer. J Gastrointest Oncol.

[29] Sargent DJ, Marsoni S, Monges G, Thibodeau SN, Labianca R, Hamilton SR (2010). Defective mismatch repair as a predictive marker for lack of efficacy of fluorouracil-based adjuvant therapy in colon cancer. J Clin Oncol.

[30] Mei WJ, Mi M, Qian J, Xiao N, Yuan Y, Ding PR (2022). Clinicopathological characteristics of high microsatellite instability/mismatch repair-deficient colorectal cancer: A narrative review. Front Immunol.

[31] Hines RB, Bimali M, Johnson AM, Bayakly AR, Collins TC (2016). Prevalence and survival benefit of adjuvant chemotherapy in stage III colon cancer patients: Comparison of overall and age-stratified results by multivariable modeling and propensity score methodology in a population-based cohort. Cancer Epidemiol.

[32] Casadaban L, Rauscher G, Aklilu M, Villenes D, Freels S, Maker AV (2016). Adjuvant chemotherapy is associated with improved survival in patients with stage II colon cancer. Cancer.

[33] Hajirawala LN, Yi Y, Herritt BC, Laurent ME, Klinger AL, Orangio GR (2023). Multiple High-Risk Features for Stage II Colon Carcinoma Portends Worse Survival Than Stage III Disease. Dis Colon Rectum.

[34] Sadahiro S, Sakamoto K, Tsuchiya T, Takahashi T, Ohge H, Sato T (2022). Prospective observational study of the efficacy of oral uracil and tegafur plus leucovorin for stage II colon cancer with risk factors for recurrence using propensity score matching (JFMC46-1201). BMC Cancer.

[35] Enofe N, Morris AD, Liu Y, Liang W, Wu CS, Sullivan PS (2020). Receipt of Adjuvant Chemotherapy in Stage II Colon Cancer and Overall Survival: A National Cancer Database Study. J Surg Res.

[36] Kumar A, Kennecke HF, Renouf DJ, Lim HJ, Gill S, Woods R (2015). Adjuvant chemotherapy use and outcomes of patients with high-risk versus low-risk stage II colon cancer. Cancer.

