# Supplementary material for: Influence of High-Risk Pathological Factors and their Interaction on the Survival Benefit of Adjuvant Chemotherapy in Stage II Rectal Cancer: A Retrospective Study
Source: J Cancer. 2024 May 5;15(11):3531–8. doi: 10.7150/jca.95769 (PMC11134436; doi:10.7150/jca.95769)
Supplement: Supplementary file 1 — Supplementary figures. [file jcav15p3531s1.pdf]

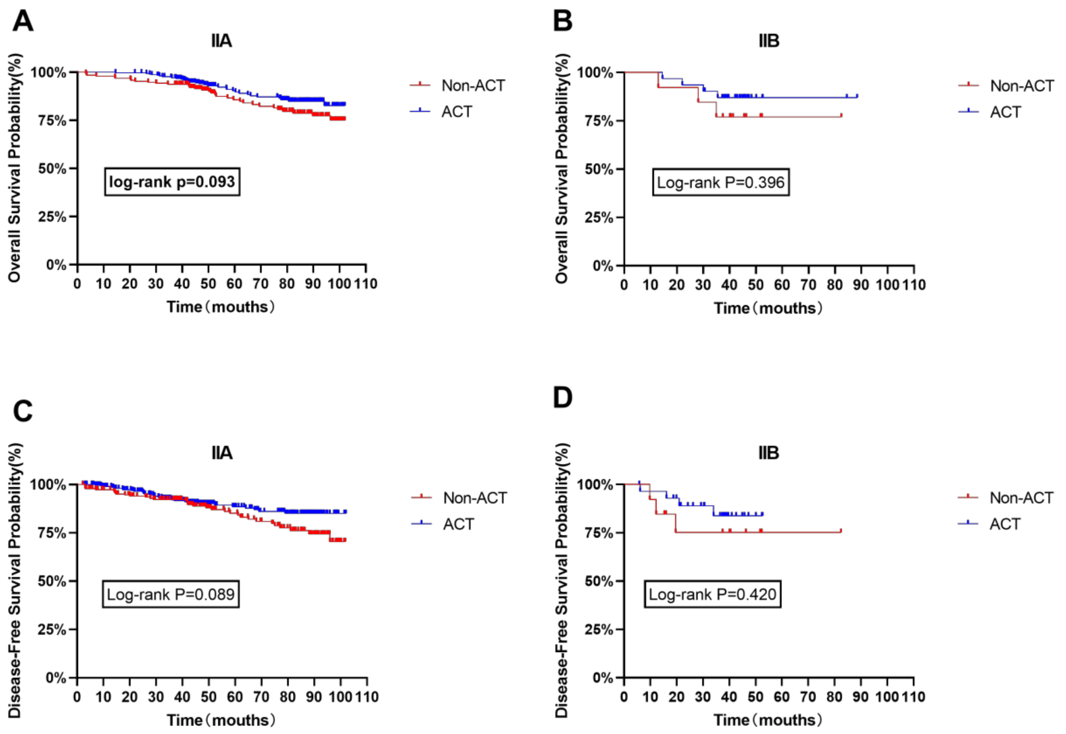

Figure S1. Disease-free survival curves of the subpopulation were calculated separately according to Kaplan-Meier method and the use of ACT in patients with different pathological TNM stages

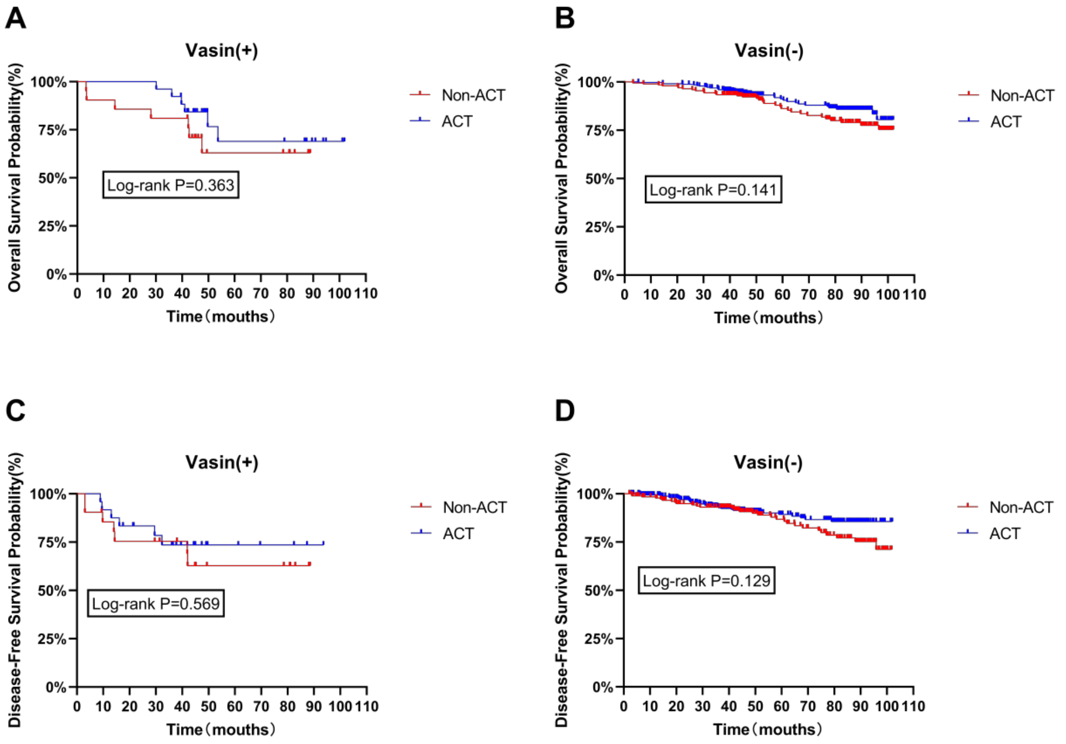

Figure S2. Disease-free survival curves for subpopulations were calculated separately according to Kaplan-Meier method, and according to the use of ACT in patients with different VNI status
